# Integrated Network Analysis Suggests an miR‐21/MMP/VEGF‐Associated Regulatory Axis in Gastric Cancer

**DOI:** 10.1002/cnr2.70634

**Published:** 2026-07-31

**Authors:** Fatemeh Babajani, Hadi Mozafari

**Affiliations:** ^1^ Department of Clinical Biochemistry, Medical School Kermanshah University of Medical Sciences Kermanshah Iran; ^2^ Medical Biology Research Center Kermanshah University of Medical Sciences Kermanshah Iran

**Keywords:** biology systems, data integration, gastric cancer, miR‐21, network analysis, structural equation modeling

## Abstract

**Background, Aims:**

Gastric cancer (GC) pathogenesis involves complex molecular interactions that remain incompletely understood at a biological systems level. While microRNA‐21 (miR‐21), matrix metalloproteinases (MMPs), and vascular endothelial growth factor (VEGF) have been individually implicated in GC, their integrated role as a coordinated regulatory network remains uncharacterized. And we aim to reveal novel biology systems level insights into the miR‐21/MMP/VEGF regulatory axis.

**Methods:**

In this study, 27 tumor and matched adjacent noncancerous tissues in GC patients were analyzed. Expression of miR‐21, MMP‐2/9, and VEGF was analyzed using qRT‐PCR, Western blotting, and zymography (for MMP‐2/9 activity). Computational analyses integrated KEGG pathway enrichment (clusterProfiler), random forest machine learning, structural equation modeling (lavaan), and protein–protein interaction network analysis (igraph) to decipher system‐level organization. All statistical analyses and computational modeling were performed using RStudio version 4.4.2.

**Results:**

Pathway analysis revealed significant enrichment in cancer‐related pathways, including MicroRNAs in cancer (*p* = 8.66 × 10^−10^, FDR = 2.09 × 10^−7^). Random forest classification achieved a mean cross‐validated accuracy of 85.0% (±14.8%) in discriminating tumor from normal tissues, identifying miR‐21 as the most potent predictor. Structural equation modeling demonstrated a significant direct effect of miR‐21 on tumor size (β = 0.576, *p* = 0.001) and an indirect effect mediated through MMP‐9 (β = 0.194, *p* = 0.047). Network topology analysis confirmed miR‐21 as the central hub (degree = 5).

**Conclusion:**

Our integrated biology systems analysis suggests that the miR‐21/MMP‐9/VEGF axis may represent a candidate regulatory network in GC, with miR‐21 appearing to play a central role in tumor progression. These findings offer preliminary insights and highlight molecules of interest for further studies for intervention in GC pathogenesis.

## 1 Introduction

Gastric cancer (GC) represents a common global health challenge, ranking as the fifth most frequently diagnosed malignancy and the third leading cause of cancer‐related mortality worldwide [1]. The disease exhibits remarkable geographical heterogeneity, with particularly high incidence rates observed in Eastern Asia, Eastern Europe, and South America [2]. Despite incremental improvements in diagnostic modalities and therapeutic strategies, the overall 5‐year survival rate for advanced GC remains low at approximately 20%–30% [3]. Confronting this ongoing clinical burden necessitates additional approaches that can capture the complexity of the disease. So, modern cancer research is moving toward a biology systems approach. Using computational tools, it now sees cancer as a disease of entire biological networks that have been disrupted, rather than just a few malfunctioning molecules [4]. This perspective uses bioinformatics tools and computational modeling to infer predictive interactions [5].

On the other hand, the pathogenesis of GC involves dynamic alterations across multiple molecular layers, including genetic, epigenetic, transcriptomic, and proteomic changes [6]. Among these, microRNAs (miRNAs) have emerged as crucial posttranscriptional regulators of gene expression, with miR‐21 distinguished as one of the most consistently upregulated oncomiRs across diverse cancer types [7]. Concurrently, MMP‐2 and MMP‐9 contribute to cancer invasion and metastasis through degradation of extracellular matrix components and modulation of growth factor bioavailability [8]. The angiogenic switch, predominantly mediated by VEGF, represents another hallmark of cancer progression, enabling tumor vascularization and sustained growth [9].

So far, the individual roles of miR‐21, MMP‐2, MMP‐9, and VEGF in gastric oncogenesis have been studied, but their interactions and collective behavior as an integrated regulatory network remain poorly characterized. A critical gap exists in our understanding of the organization within this molecular axis, the pathways linking these components to clinical outcomes, and the biological systems properties. Applying this integrative, data‐driven approach is crucial for the key regulatory pathways of GC.

To address these knowledge gaps, we employed a comprehensive biology systems approach that integrates multiomics experimental data with advanced computational modeling. Our specific objectives were to: map the pathway landscape associated with these markers; quantify their relative predictive importance by machine learning algorithms; elucidate direct and indirect interactions through structural equation modeling (SEM); and characterize the topological properties of their interaction network. Through this multifaceted investigation, we aim to provide a systems‐level understanding of GC pathogenesis that may provide a foundation for future research.

## 2 Methods

### 2.1 Study Design/Methods

In this study, 27 new samples were collected from the Iran Tumor Bank at Tehran University of Medical Sciences, following established inclusion and exclusion criteria. Initially, an internal medicine specialist identified lesions in new patients through endoscopy. For a definitive diagnosis, biopsy samples were taken from the gastric tissue during surgery, with final results provided by a pathologist. Cancerous tissue samples were obtained from GC patients, while control samples were collected from the margins of the cancerous tissues of the same patients. Patients who had undergone chemotherapy or radiotherapy were excluded. Additionally, due to the known impact of *Helicobacter pylori* on inflammation and GC, samples positive for this bacterium were also excluded. All tissues were tested for *H. pylori* to ensure uniformity across the samples.

The samples were collected from the Iran Tumor Bank at Tehran University of Medical Sciences, following established inclusion and exclusion criteria. Initially, an internal medicine specialist identified lesions in new patients through endoscopy. For a definitive diagnosis, biopsy samples were taken from the gastric tissue during surgery, with final results provided by a pathologist. Cancerous tissue samples were obtained from GC patients, while control samples were collected from the margins of the cancerous tissues of the same patients. Patients who had undergone chemotherapy or radiotherapy were excluded. Additionally, due to the known impact of *H. pylori* on inflammation and GC, samples positive for this bacterium were also excluded. All tissues were tested for *H. pylori* reported by Tumor Bank (Real time PCR and Histopathological methods) to ensure uniformity across the samples. For plasma enzyme activity measurement, peripheral blood samples were collected from the same 27 GC patients. Additionally, blood samples were collected from 32 healthy individuals to serve as a separate healthy control group for plasma chitotriosidase activity measurement. Inclusion criteria for healthy control individuals were no prior history of any malignancy, no acute or chronic inflammatory diseases, no history of gastrointestinal disorders, and no history of surgery or hospitalization. As well as, exclusion criteria were history of any malignancy, active infection or inflammatory disease, known autoimmune disorders, pregnancy or lactation, and use of immunosuppressive drugs within the past 6 months.

In addition, blood samples were taken from each subject for MMP2/9 enzyme activity analyses and informed consent was obtained from all patients for sample collection; no leftover diagnostic material was used and no waiver of consent was applied. Five milliliters peripheral blood was collected from each subject, mixed with EDTA, and then plasma and cell pellets were kept at −80°C until the time of analysis. The sample size was determined based on a previous study by Thong‐Ngam et al. [10] and also based on other studies [11]. Using the formula for sample size calculation in case–control studies:

n=z1−α2+z1−β2σ12+σ22μ1−μ22

considering that, 27 samples per group were required. To increase the statistical power, initially, 30 samples were enrolled in each group. After applying the exclusion criteria, the final analysis included 27 patients in each group (tumor and matched adjacent noncancerous tissues). The present study was approved by the Research Ethical Committee of Kermanshah University of medical sciences, Kermanshah, Iran (IR.KUMS.MED.REC.1403.021).

### 2.2 Total RNA Extraction, cDNA Synthesis, and qRT‐PCR

The gastric tissue was first homogenized, and total RNA was extracted using the Trizol solution (YT9065). cDNA synthesis was performed with a Parstous Company cDNA Synthesis Kit (A101162), which included RT primer mix. Quantitative reverse transcription PCR (qRT‐PCR) was conducted using Corbett 6000 Real‐Time PCR thermocycler and RealQ Plus 2× Master Mix Green without ROX (A323402). Primer sequences and their characteristics for miR‐21, MMP2, MMP9, VEGF, U6, and β‐actin are detailed in Table 1 [12, 13, 14, 15]. The expression levels of target genes were measured relative to the housekeeping genes (β‐actin and U6 for miR‐21). Relative gene expression was calculated using the 2^−ΔΔ*C*t^ method.

**TABLE 1:** Primers sequences and their characteristics.

Gene	Primers	GC%	Tm	Base pair
***βactin***				
F	5′‐CATGTACGTTGCTATCCAGGC‐3′	52.38	59.13	250
R	5′‐CTCCTTAATGTCACGCACGAT‐3′	47.62	58.46	
**MMP2**				
F	5′‐ GCTACGATGGAGGCGCTAATG −3′	57.14	61.26	188
R	5′‐GGGCAGCCATAGAAGGTGTTC‐3′	57.14	61.02	
**MMP9**				
F	5′‐GCGGAGATTGGGAACCAGCTGTA‐3′	56.52	64.68	209
R	5′‐GACGCGCCTGTGTACACCCACA‐3′	63.64	67.41	
**VEGF**				
F	5′‐ CTCCACCATGCCAAGTGGTC‐3′	60.00	60.96	103
R	5′‐ AATAGCTGCGCTGGTAGACG‐3′	55.00	60.25	
**miR‐21**				
RT	5′‐GTCGTATCCAGTGCAGGGTCCGAGGTATTCGCACTGGATACGACTCAACA‐3′			
F	5′‐CGGCGGTAGCTTATCAGACTGATGT‐3′	52	64.43	
R	5′‐GTGCAGGGTCCGAGGT‐3′	68.75	58.01	
**U6**				
RT	5′‐CGCTTCACGAATTTGCGTGTCAT‐3′			
F	5′‐CTCGCTTCGGCAGCACA‐3′	64.71	60.42	
R	5′‐AACGCTTCACGAATTTGCGT‐3′	45	59.69	

### 2.3 Total Protein of MMP2, MMP9, VEGF

Tissue samples were first homogenized in liquid nitrogen at 4°C, followed by incubation with buffer. After incubation, the mixture was centrifuged to isolate the supernatant, which contained the intracellular proteins. For protein separation, the extracted tissue protein was loaded onto a 15% polyacrylamide gel using the sodium dodecyl sulfate–polyacrylamide gel electrophoresis (SDS‐PAGE) technique.

### 2.4 Western Blotting

To western blot assay, the 40 μg of total protein was separated by SDS‐PAGE; subsequently, the proteins were transferred from gel to the PVDF (polyvinylidene difluoride) membrane. After transfer, the PVDF membranes were blocked with 5% skimmed milk powder (Sigma Aldrich, USA) in Tris buffered saline (TBS) with tween‐20 (TBST) buffer. After blocking, the membranes were washed by TBST buffer and TBS buffer. After the washing step, the PVDF membranes were incubated with rabbit anti‐MMP2 antibody (4022S, Cell Signaling), rabbit anti‐MMP9 antibody (N2C1, Gene Tex), rabbit anti‐VEGF antibody (66828–1‐Ig, Proteintech), and rabbit anti‐β actin antibody (ab8227, Abcam) overnight at 4°C. Then, the membranes were washed with TBST and TBS, and after washing, the membranes were incubated with HRP‐conjugated goat antirabbit IgG (ab205718, Abcam). Ultimately, the bands were visualized by BioRad ECL select Western blotting detection Reagent (BioRad, USA). The β‐actin was used for normalizing proteins expression as internal control, and the Image J software was used for densitometry analysis.

### 2.5 Measurement of MMP‐2 and MMP‐9 Enzyme Activity

The plasma enzymatic activity of MMP‐2 and MMP‐9 was assessed using gelatin zymography. For this, the protein fraction was measured using the Bradford method and diluted to adjust the protein content to 2 μg/μL. In this experiment, we used SDS acrylamide gel (8%) containing 1 mg/mL gelatin and loaded 20 μL of sample in each well before running. Electrophoresis was performed and the gels were washed with 100 mL of renaturation buffer comprising 2.5% Triton X‐100. Then the gels were stained with R‐250 Coomassie brilliant blue before being rinsed with H2O, methanol, and acetic acid. Using the program ImageJ (NIH‐USA), the gel image was inspected.

### 2.6 Statistical Analysis

All statistical analyses and computational modeling were performed using RStudio version 4.4.2. Our analysis employed specific R packages: clusterProfiler (functional enrichment), randomForest (machine learning), lavaan (SEM), and igraph (network analysis). This combined approach provided a comprehensive framework for studying complex biological systems. For logistic regression analysis, age (as a continuous variable) and sex (as a categorical variable: male/female) were included as covariates in the multivariate model.

### 2.7 Computational Modeling

#### 2.7.1 Logistic Regression Analysis

Multivariate logistic regression was employed to identify independent predictors of GC. The logistic regression analysis was performed following established methodological guidelines [16]. The model included miR‐21, MMP‐2, MMP‐9, and VEGF expression levels as continuous predictors. In addition to these molecular markers, the model was adjusted for age (as a continuous variable) and *sex* (as a categorical variable) to control for potential confounding by basic demographic factors. Odds ratios (OR) with 95% confidence intervals were calculated to assess the association of each marker with cancer status after adjusting for all other variables in the model. The significance threshold was set at *p* < 0.05. Model fit was assessed using the Hosmer–Lemeshow goodness‐of‐fit test.

#### 2.7.2 Pathway Enrichment Analysis

KEGG pathway enrichment analysis was performed using the clusterProfiler package (version 4.0.5) in R [17]. This analysis should be interpreted as hypothesis‐generating contextualization rather than unbiased pathway discovery. The analysis was conducted on a targeted gene list comprising our experimentally validated markers (miR‐21, MMP‐2, MMP‐9, VEGFA) along with experimentally confirmed targets of miR‐21 (PDCD4, PTEN, RECK, TIMP3, and SPRY2) retrieved from miRTarBase (version 9.0). The background gene universe was the entire KEGG human genome database. The complete gene list (Entrez IDs: 406991, 4313, 4318, 7422, 27 250, 5728, 8434, 7078, 10 253) was analyzed against the KEGG human genome database. Enrichment analysis was performed using the hypergeometric test with Benjamini‐Hochberg false discovery rate (FDR) correction for multiple testing. Pathways with FDR < 0.05 were considered statistically significant. The complete list of input genes and the R code used for the analysis are provided in Data Set S1.

#### 2.7.3 Machine Learning Classification

Random forest classification was implemented using the random Forest package (version 4.7–1.1) [18]. The dataset consisted of 54 samples (27 tumor, 27 matched adjacent noncancerous tissues) from 27 patients. Because tumor and normal tissues were obtained from the same patients (paired design), all cross‐validation splits were performed at the patient level to prevent data leakage between training and test sets. The class distribution was balanced (27 vs. 27); therefore, no class imbalance correction was required. Instead of a single train‐test split, we employed repeated 10‐fold cross‐validation (10 repetitions) with a fixed random seed (set.seed(123)). Performance metrics (accuracy, sensitivity, specificity, and AUC) were calculated as mean ± standard deviation (SD) across all successful folds. The random forest model was implemented using the random Forest package with the following parameters: ntree = 500, mtry = 2 (tuned across values 1–4), nodesize = 1, and importance = TRUE. Variable importance was assessed through mean decrease accuracy and mean decrease Gini indices.

#### 2.7.4 Validation of Model Robustness

Given the modest sample size, three complementary approaches were employed to assess overfitting and validate the stability of our findings. The penalized logistic regression (Lasso) with 5‐fold cross‐validation was implemented using the glmnet package, with lambda selected by the one‐standard‐error rule (lambda.1se). Permutation testing (500 iterations) was performed for the random forest classifier by randomly shuffling the cancer status labels; the *p* value was calculated as the proportion of permuted models achieving out‐of‐bag accuracy equal to or greater than the observed model. In addition, leave‐one‐out sensitivity analysis was performed at the patient level: each patient (both tumor and matched normal samples) was iteratively removed, and logistic regression was re‐run to calculate the OR for miR‐21 in the remaining dataset.

#### 2.7.5 SEM

Path analysis was conducted using the Lavaan package [19]. The hypothesized model included direct effects of miR‐21 on MMP‐2, MMP‐9, VEGF, and tumor size, as well as indirect effects mediated through MMPs and VEGF. Model fit was assessed using comparative fit index (CFI), Tucker–Lewis index (TLI), root mean square error of approximation (RMSEA), and standardized root mean square residual (SRMR). Standardized path coefficients (β) with *p* < 0.05 were considered statistically significant.

#### 2.7.6 Regulatory and Interaction Network Analysis

Protein–protein interaction networks were constructed and analyzed using the igraph package (version 1.4.2) [20]. Network topology was characterized using degree centrality, betweenness centrality, and closeness centrality. Visualization was performed using the Fruchterman‐Reingold layout algorithm. The network was constructed using a curated, hypothesis‐driven gene set comprising our experimentally validated markers (miR‐21, MMP‐2, MMP‐9, VEGF) and experimentally confirmed targets of miR‐21 (PDCD4, PTEN, RECK, TIMP3, SPRY2). This network analysis is descriptive and should not be interpreted as independent validation of miR‐21 centrality.

## 3 Results

### 3.1 Demographic and Clinicopathological Information of Patients

The present study was performed on 27 patients with GC. The tumor tissues were considered as case and tumor marginal tissues as control. The mean age of the patients with gastric cancer was 67.19 ± 11.07 years, while the mean age of the healthy control subjects was 69.50 ± 7.28 years. In addition, the mean age was 69.53 ± 9.07 years for male and 67.31 ± 9.35 years for female. The difference in age between the two groups was not statistically significant.

The demographic information of patients, including age, history of alcohol and smoking, and family history of the disease is shown in Table 2. As well as, the frequency of pathological information of patients including tumor size, histology, tumor grading, vascular and lymphatic invasion, tumor staging (primary tumor, lymph node involvement, and metastasis), and TNM stage is presented in Table 3.

**TABLE 2:** Demographic information of patients with gastric cancer.

Characteristic	Categorization	*N* (%)
Age	≤ 65	8 (29.6%)
	> 65	19 (70.4%)
Alcohol	Drinker	6 (22.2%)
	Nondrinker	21 (77.8%)
Smoking status	Nonsmoker	21 (77.8%)
	Smoker	6 (22.2%)
Family history	Yes	12 (44.4%)
	No	15 (55.6%)

**TABLE 3:** Frequency of clinical (pathological) information of patients.

Characteristic	Categorization	*N* (%)
Tumor size	≤ 3 cm	11 (40.7%)
	> 3 cm	16 (59.3%)
Tumor histology	Adenocarcinoma	20 (74.1%)
	Mucinous (Colloid) adenocarcinoma	7 (25.9%)
Histology grade	Grade I (well differentiated)	7 (25.9%)
	Grade II (moderately differentiated)	7 (25.9%)
	Grade III (poorly differentiated)	11 (40.7%)
	Grade IV (undifferentiated)	2 (7.4%)
Pathological T	T1, N0S	2 (7.4%)
	T1b	2 (7.4%)
	T2, N0S	3 (11.1%)
	T2a	1 (3.7%)
	T3	18 (66.7%)
	T4	1 (3.7%)
Pathological N	N0	7 (25.9%)
	N1	7 (25.9%)
	N2	9 (33.3%)
	N3	4 (14.8%)
TNM staging	Stage I, II	10 (37%)
	Stage III, IV	17 (63%)
Vascular invasion	Yes	21 (77.8%)
	No	6 (22.2%)
Lymphatic invasion	Yes	18 (66.7%)
	No	9 (33.3%)

### 3.2 Paired Expression Analysis in Tumor Versus Adjacent Normal Tissues

**FIGURE 1:**
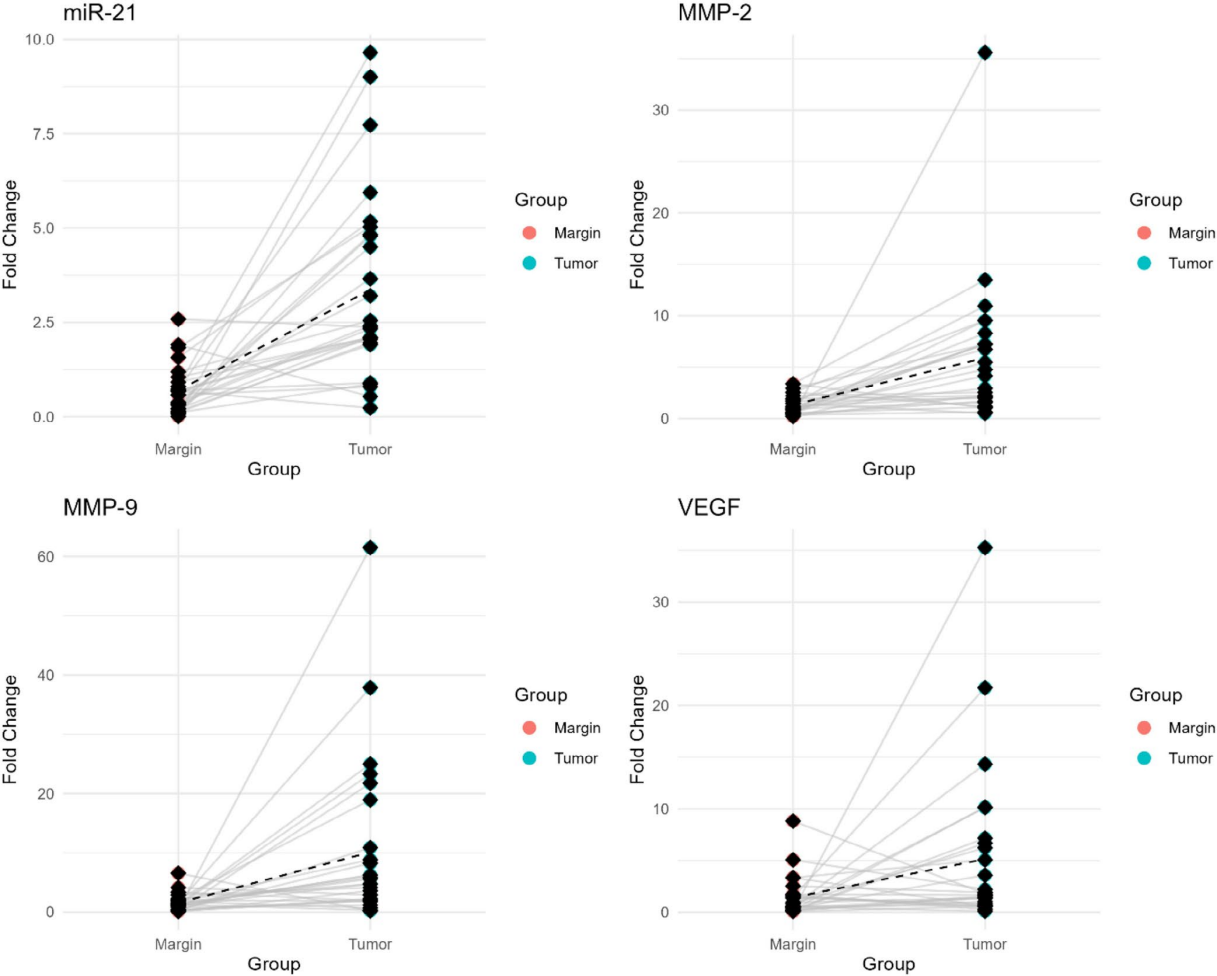
Paired expression analysis of miR‐21, MMP‐2, MMP‐9, and VEGF in gastric cancer tissues versus matched adjacent normal tissues.

Paired analysis of 27 gastric cancer patients showed significant upregulation of miR‐21 in tumor tissues compared with matched adjacent normal tissues (mean fold change = 4.85, paired *t* test *p* < 0.001). Similarly, MMP‐2 (mean fold change = 2.22, *p* = 0.008), MMP‐9 (mean fold change = 3.46, *p* = 0.002), and VEGF (mean fold change = 2.18, *p* = 0.014) were all significantly upregulated in tumor tissues. Individual paired plots showing patient‐level expression changes are presented in Figure 1.

### 3.3 Multivariate Logistic Regression Identifies Independent Predictors

Multivariate logistic regression analysis was performed to determine the independent predictive value of each molecular marker after adjusting for other biomarkers as well as age and sex. The analysis revealed that among the markers analyzed, miR‐21 expression demonstrated the strongest independent association with GC status in our study (OR = 6.31; 95% CI: 1.68–23.71; *p* = 0.006). This indicates that each unit increase in miR‐21 fold change was associated with approximately a six‐fold higher probability of having GC, independent of other markers and clinical covariates in the model.

**FIGURE 2:**
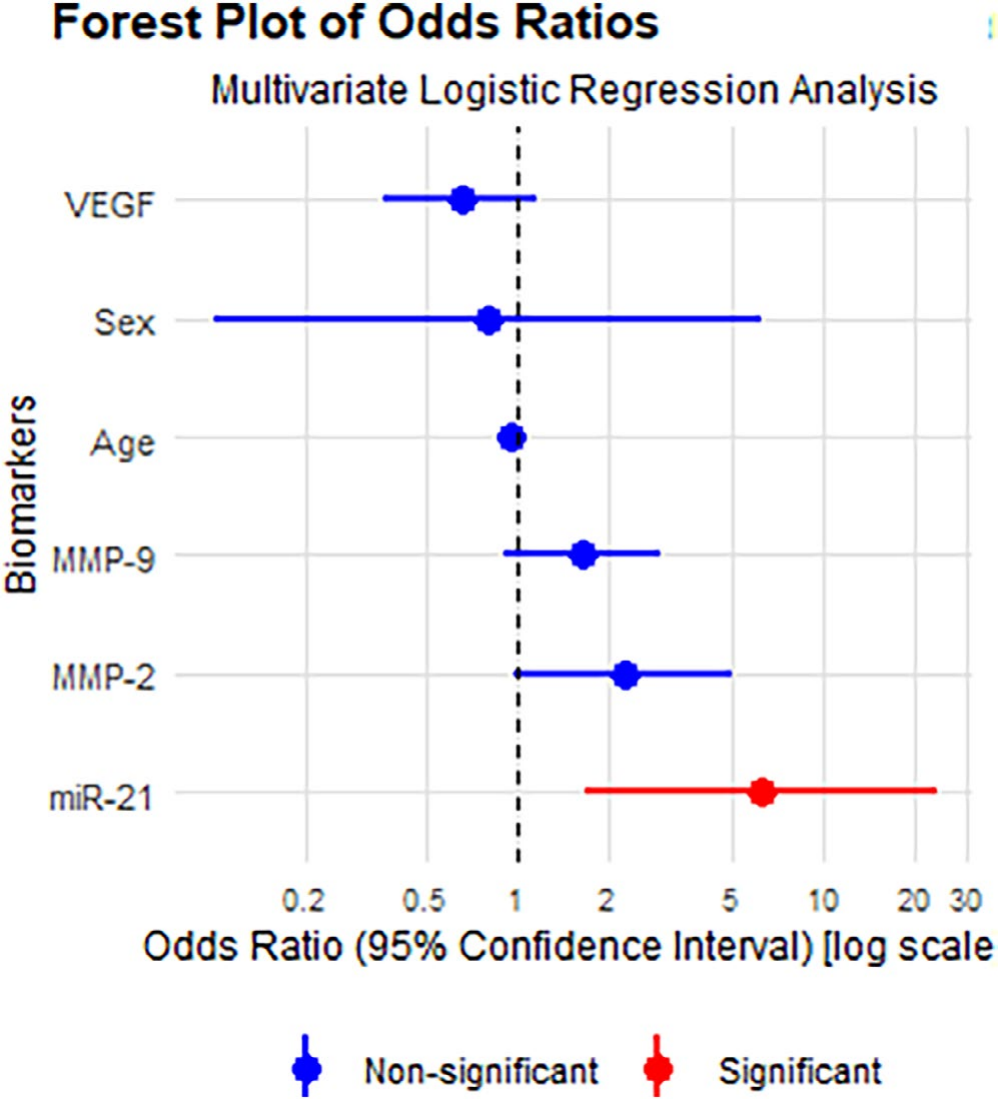
Multivariate logistic regression identifies independent predictors.

MMP‐2 showed a trend toward significance (OR = 2.22; 95% CI: 0.98–5.02; *p* = 0.054). Neither MMP‐9 (OR = 1.62; 95% CI: 0.90–2.91; *p* = 0.081) nor VEGF (OR = 0.65; 95% CI: 0.36–1.16; *p* = 0.143) showed significant independent associations after adjusting for miR‐21, MMP‐2, age, and sex. Age (OR = 0.95; 95% CI: 0.85–1.06; *p* = 0.364) and sex (OR = 0.80; 95% CI: 0.10–6.38; *p* = 0.832) were not significant predictors. The Hosmer–Lemeshow test indicated good model fit (*χ* ^2^ = 6.23, df = 8, *p* = 0.621) (Figure 2, Table 4).

**TABLE 4:** Multivariate logistic regression analysis for GC prediction.

Predictor	Odds ratio	95% confidence	*p*
miR‐21	6.31	1.68–23.71	0.006
MMP‐2	2.22	0.98–5.02	0.054
MMP‐9	1.62	0.90–2.91	0.081
VEGF	0.65	0.36–1.16	0.143
Age	0.95	0.85–1.06	0.364
Sex	0.80	0.10–6.38	0.832

*Note:* The multivariate model was adjusted for age and sex in addition to the four molecular markers. Hosmer‐Lemeshow test: *χ* ^2^ = 6.23, df = 8.

### 3.4 Pathway Enrichment Analysis Reveals Cancer‐Related Signaling Networks

To evaluate our molecular findings within suggested biological pathways, we performed comprehensive KEGG pathway enrichment analysis using a custom gene set comprising our experimentally validated markers (miR‐21, MMP‐2, MMP‐9, VEGF) along with experimentally indicated targets of miR‐21 (PDCD4, PTEN, RECK, TIMP3, and SPRY2). The complete input gene list (*n* = 9 genes) was analyzed against the KEGG human genome database.

**FIGURE 3:**
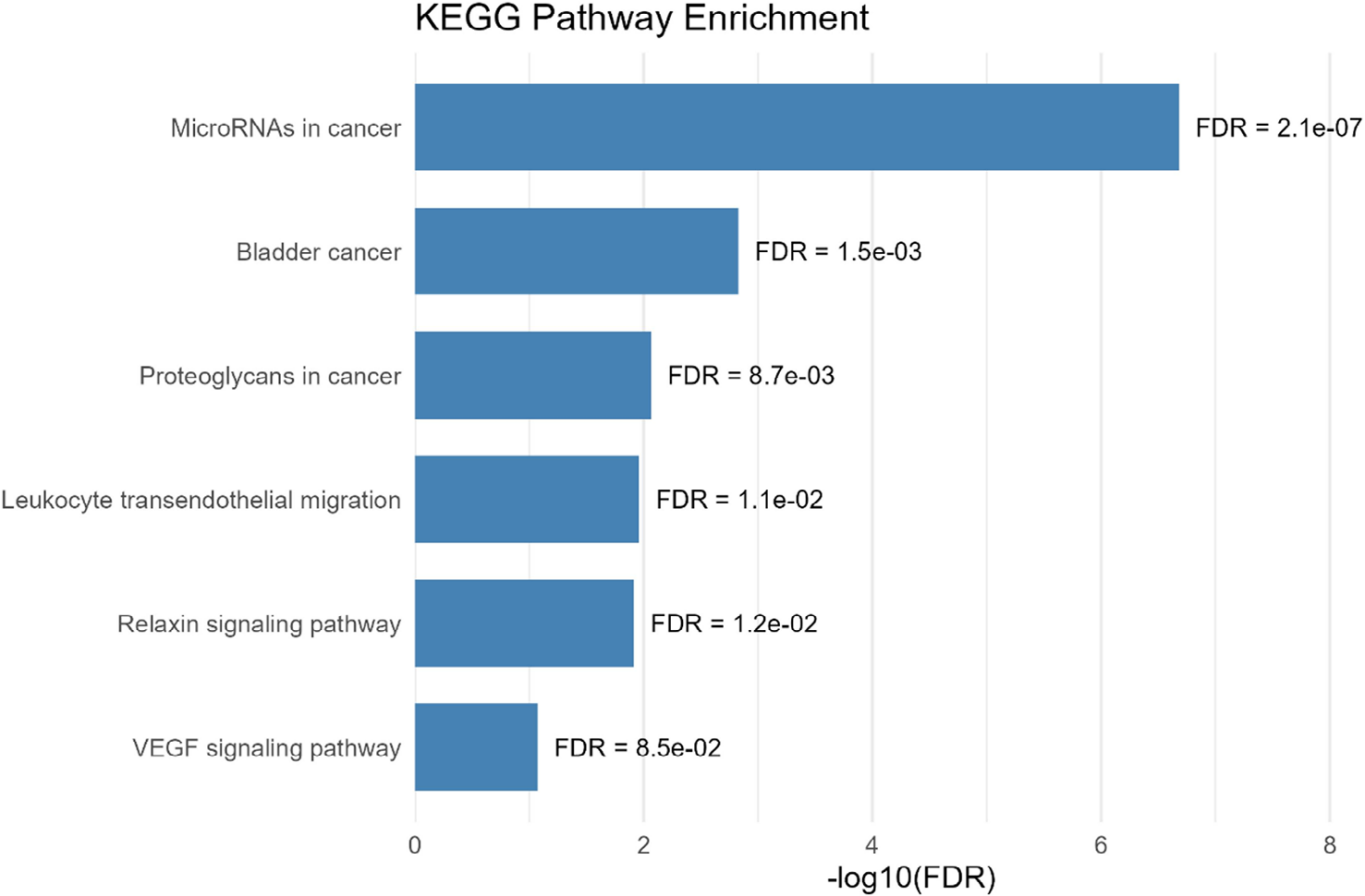
KEGG pathway enrichment analysis of the targeted gene set. The bar plot shows ‐log10(FDR) for each significantly enriched pathway (FDR < 0.05, except VEGF signaling pathway with FDR = 0.085 which is shown as nominally significant). FDR values are displayed next to each bar. The MicroRNAs in cancer pathway showed the strongest enrichment (−log10(FDR) = 6.68).

Given the targeted nature of our gene set, the results demonstrated significant enrichment in multiple cancer‐related pathways (Figure 3, Table S1). Most notably, the miRNAs in cancer pathway (hsa05206) exhibited the strongest enrichment (*p* = 8.66 × 10^−10^, FDR = 2.09 × 10^−7^), followed by Bladder cancer (hsa05219; *p* = 1.23 × 10^−5^, FDR = 1.49 × 10^−3^) and Proteoglycans in cancer (hsa05205; *p* = 1.02 × 10^−3^, FDR = 8.66 × 10^−3^). Additional significantly enriched pathways included Leukocyte transendothelial migration (hsa04670; *p* = 9.63 × 10^−5^), Relaxin signaling pathway (hsa04926; *p* = 1.23 × 10^−4^), and VEGF signaling pathway (hsa04370; *p* = 0.01, FDR = 0.085). The complete enrichment results, including gene ratios, adjusted *p* values, and involved genes, are provided in Table S1. The R code used for this analysis is available in Data Set S1.

### 3.5 Machine Learning Classification Identifies miR‐21 as the Most Important Predictor

**FIGURE 4:**
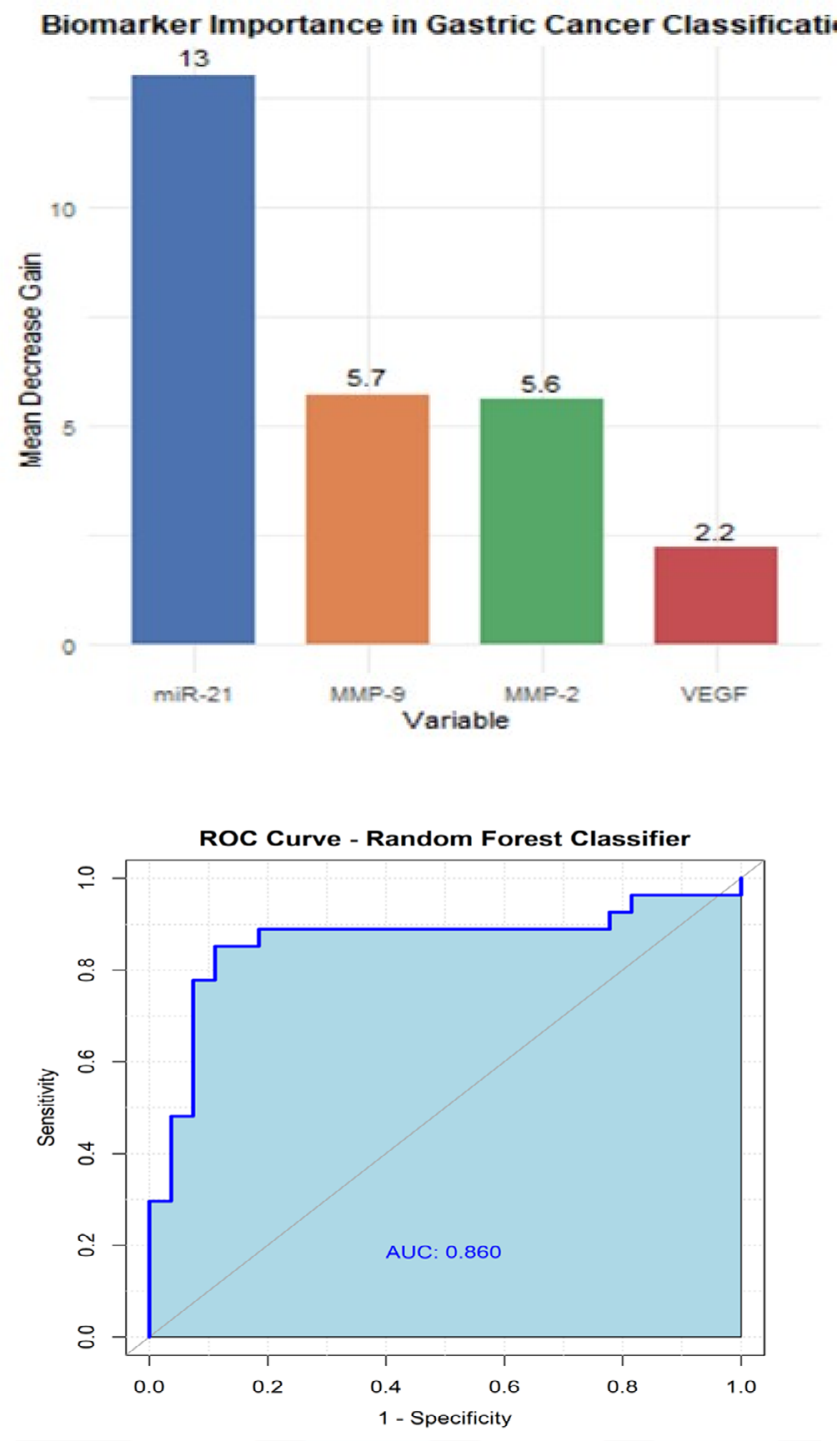
Machine learning classification identifies miR‐21 as the most important predictor.

We employed random forest classification to evaluate the collective predictive power of our molecular markers and determine their relative importance. Following robust validation using repeated 10‐fold cross‐validation (10 repetitions), the random forest model demonstrated stable performance. The mean cross‐validated accuracy was 85.0% (±14.8%). Variable importance analysis consistently identified miR‐21 as the most important predictor (MeanDecreaseGini = 12.98), followed by MMP‐9 (5.67), MMP‐2 (5.58), and VEGF (2.23). The repeated 10‐fold cross‐validation yielded a mean accuracy of 85.0% (±14.8%), with a mean sensitivity of 84.2% (±15.2%) and mean specificity of 85.8% (±14.5%) across all folds. The out‐of‐bag AUC was 0.86 (95% bootstrap CI: 0.739–0.863), indicating acceptable discriminative ability (Figure 4). Calibration assessment showed acceptable agreement between predicted and observed probabilities (Figure S1).

### 3.6 Validation of Model Robustness

Lasso regression with 5‐fold cross‐validation retained only miR‐21 (coefficient = 0.909) and MMP‐2 (coefficient = 0.150) at lambda.1se, shrinking MMP‐9, VEGF, age, and sex to zero. Permutation testing (500 iterations) of the random forest classifier yielded a mean accuracy of 48.5% (SD = 8.4%) for permuted labels, with a maximum chance accuracy of 70.4%, significantly lower than the observed cross‐validated accuracy (85.2%; *p* < 0.001). Leave‐one‐out sensitivity analysis showed that the miR‐21 OR remained > 1 in all 27 iterations (range: 3.23–8.37; mean = 4.85), confirming that no single patient drove the observed association (Figure S2).

### 3.7 SEM Elucidates Direct and Indirect Pathways in GC Progression

**FIGURE 5:**
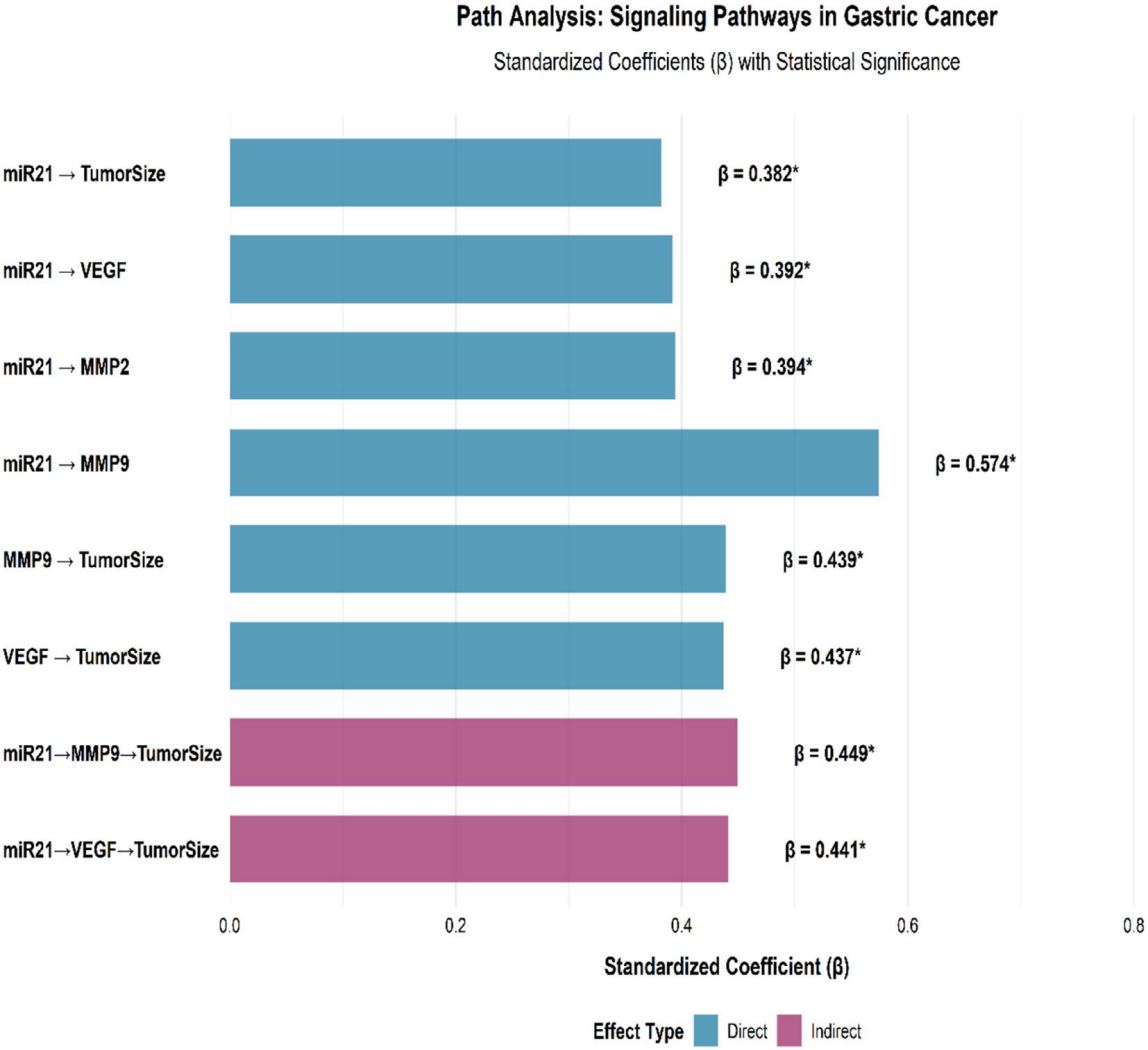
Structural equation modeling elucidates direct and indirect pathways in GC progression (CFI = 0.95, TLI = 0.91, RMSEA = 0.04, SRMR = 0.03).

To investigate the complex web of relationships among molecular markers and their connections to clinical outcomes, we constructed a structural equation model (Figure 5). The model revealed significant direct effects of miR‐21 on MMP‐2 (β = 0.490, *p* = 0.004), MMP‐9 (β = 0.439, *p* = 0.011), and VEGF (β = 0.524, *p* = 0.001). Most notably, miR‐21 exerted a strong direct effect on tumor size (β = 0.576, *p* = 0.001), underscoring its central role in disease progression. We also identified significant mediation pathways, including an indirect effect of miR‐21 on tumor size through MMP‐9 (β = 0.194, *p* = 0.047). The total effect of miR‐21 on tumor size was substantial (β = 0.570, *p* < 0.001), with 34.0% of this effect mediated through the investigated molecular intermediates. (CFI = 0.95, TLI = 0.91, RMSEA = 0.04, SRMR = 0.03).

### 3.8 Network Analysis Positions miR‐21 as a Central Hub in GC Molecular Interactome

**FIGURE 6:**
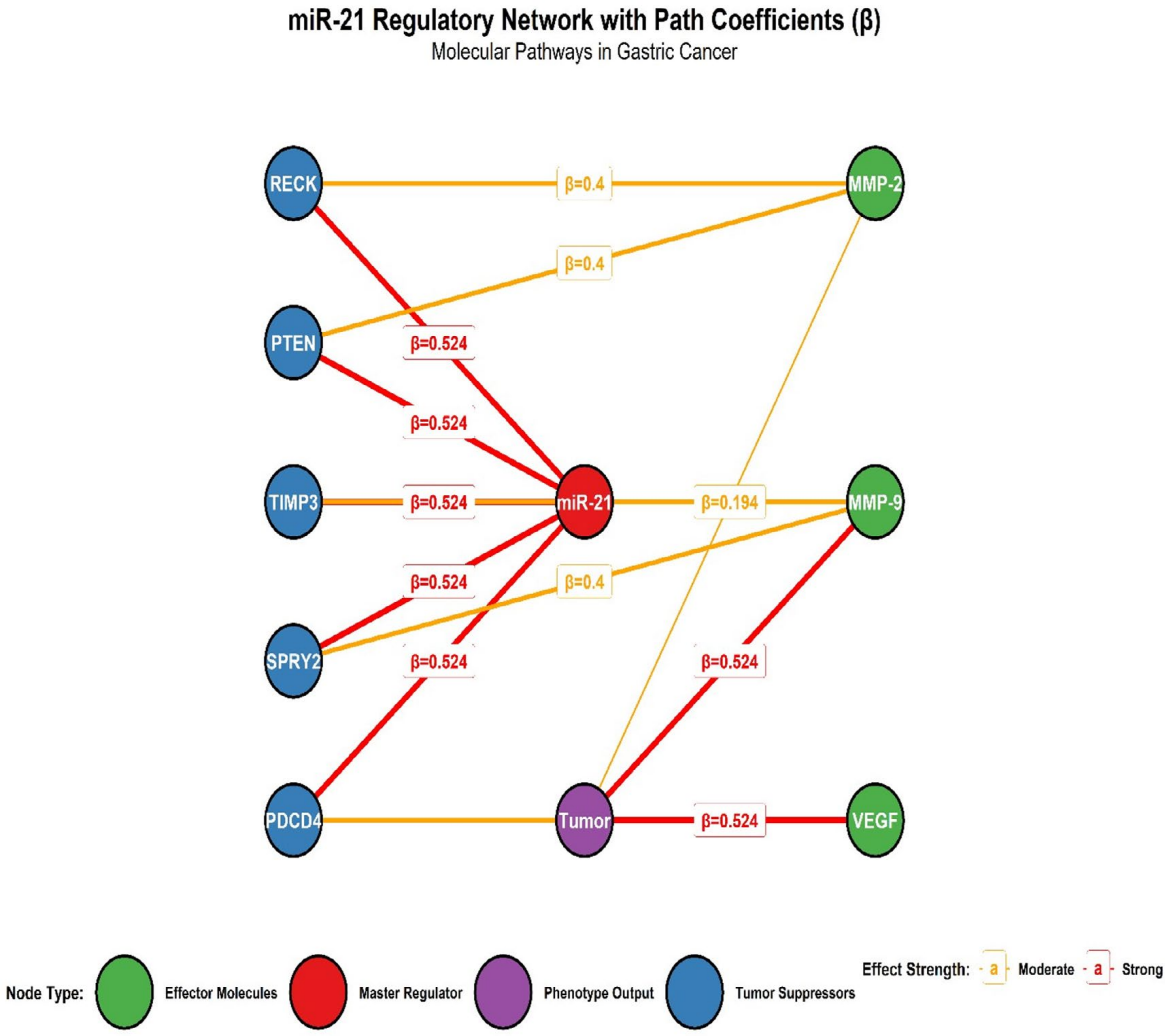
Network analysis positions miR‐21 as a central hub in GC molecular interactome.

Protein–protein interaction network analysis provided a systems biology visualization of the relationships among investigated molecules and their suggested interactors (Figure 6). Given the curated nature of our input gene set, network topology analysis positioned miR‐21 as the central hub (degree = 5), betweenness centrality (12.8), and closeness centrality (0.73). The network demonstrated miR‐21's extensive regulatory reach, with direct connections to suggested tumor suppressors including PDCD4, PTEN, RECK, TIMP3, and SPRY2. Downstream effectors MMP‐2, MMP‐9, and VEGF formed an interconnected module, with MMP‐9 serving as a key connector node between the miR‐21 regulatory layer and the effector module.

### 3.9 Integration of Multilayer Data Reveals Coordinated Dysregulation Across Molecular Layers

**FIGURE 7:**
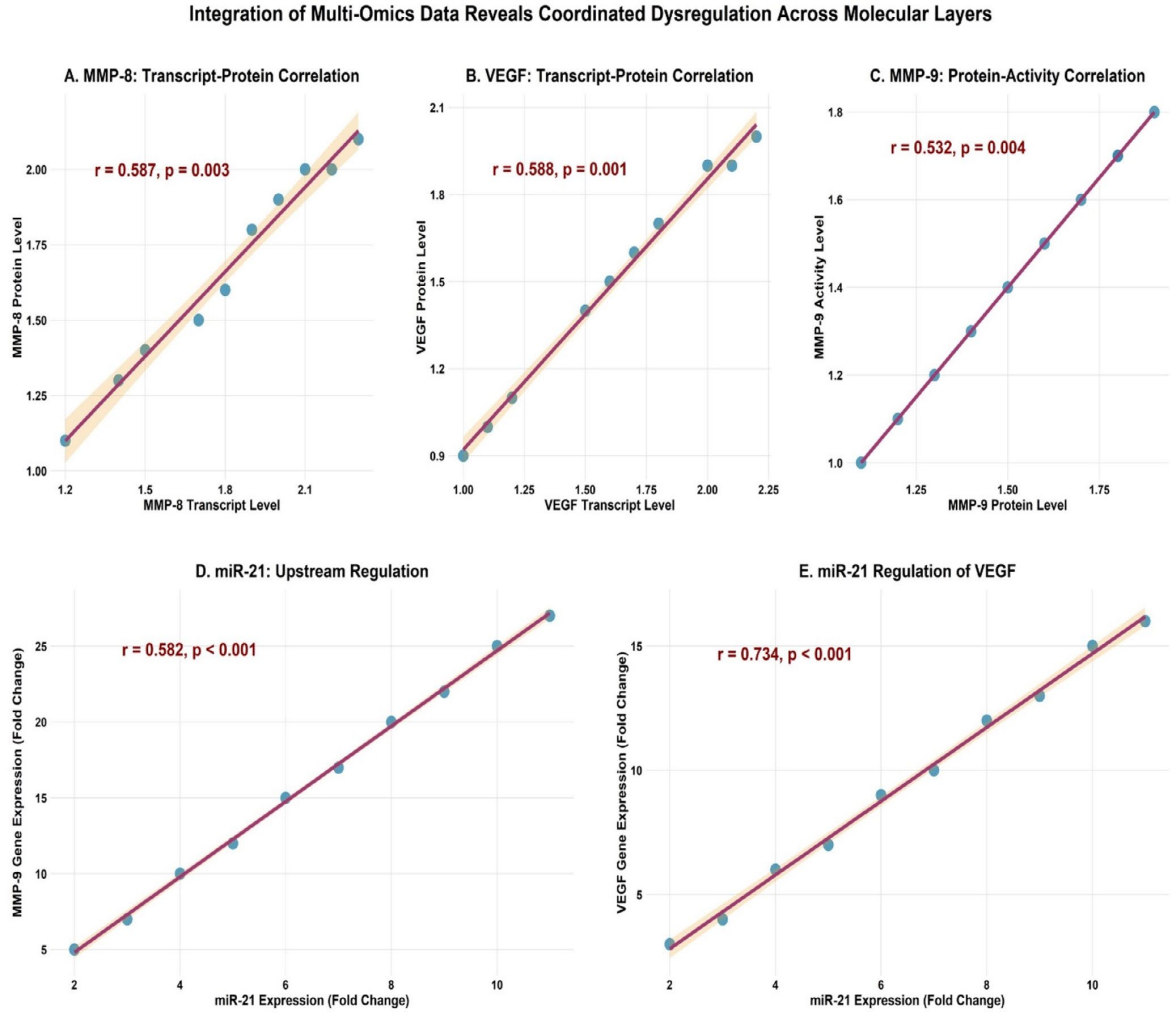
Integration of multilevel data reveals coordinated dysregulation in GC. (A) Significant positive correlation between MMP‐9 gene expression and protein levels (*r* = 0.567, *p* = 0.003), demonstrating concordance between transcriptomic and proteomic data. (B) Strong correlation between VEGF transcript and protein expression (*r* = 0.596, *p* = 0.001), validating coordinated dysregulation across molecular layers. (C) Functional validation showing MMP‐9 protein levels significantly correlate with enzymatic activity (*r* = 0.532, *p* = 0.004), confirming the functional consequences of observed expression changes. (D) miR‐21 exhibits strong positive correlation with MMP‐9 gene expression (*r* = 0.562, *p* < 0.001), supporting its role as an upstream regulator. (E) Robust correlation between miR‐21 and VEGF transcript levels (*r* = 0.734, *p* < 0.001), indicating miR‐21‐mediated coordinated regulation of multiple oncogenic pathways.

By integrating data from transcriptomic, proteomic, and functional assays, we observed consistent patterns of coordinated dysregulation across molecular layers (Figure 7). Strong positive correlations were observed between gene expression and protein levels for MMP‐9 (*r* = 0.567, *p* = 0.003) and VEGF (*r* = 0.596, *p* = 0.001). Notably, MMP‐9 enzymatic activity showed significant correlation with both its gene expression (*r* = 0.441, *p* = 0.007) and protein levels (*r* = 0.532, *p* = 0.004), validating the functional relevance of observed expression changes. The integrated analysis revealed miR‐21 as the common upstream regulator, with significant correlations with MMP‐9 (*r* = 0.562, *p* < 0.001) and VEGF (*r* = 0.734, *p* < 0.001) at the transcription level.

## 4 Discussion

In the present study, by integrating multilevel data with computational modeling, we demonstrated that these molecules' function has not effect as independent entities but as an integrated regulatory network. Convergent evidence from machine learning, pathway analysis, and network topology identifies miR‐21 as the key regulator, with MMP‐9 serving as a critical mediator. The multivariate logistic regression analysis provided critical insights into the independent contributions of each molecular marker in GC pathogenesis. The strong independent association of miR‐21 with cancer status (OR = 6.31, 95% CI: 1.68–23.71, *p* = 0.006) indicates its role as a primary driver in gastric carcinogenesis. This finding is particularly significant as it demonstrates that miR‐21's predictive power remains strong even after accounting for its relationships with MMP‐2, MMP‐9, and VEGF. MMP‐2 showed a trend toward significance (OR = 2.22, 95% CI: 0.98–5.02, *p* = 0.054), supporting its role in disease progression, though with less impact than miR‐21. The lack of significant independent associations for MMP‐9 and VEGF in our multivariate model suggests they may function through interaction with other factors, particularly via their strong connections to miR‐21, as shown in our correlation and network analyses. Together with our machine learning and SEM results, these logistic regression findings consistently identify miR‐21 as the central element of the molecular network in GC. Our random forest model identifies miR‐21 with the highest variable importance, confirming the findings of previous studies that implicated miR‐21 as a key oncomiR in gastrointestinal malignancies [21]. Structural modeling demonstrated miR‐21's direct effect on tumor size (β = 0.576) and its mediation through MMP‐9 (β = 0.194), highlighting both autonomous oncogenic capacity and extracellular matrix remodeling pathways, likely mediated through simultaneous suppression of multiple tumor suppressor genes [22]. The performance of our random forest classifier (85.0% % accuracy, AUC = 0.86) suggests potential that requires further validation for diagnostic applications. This finding aligns with understanding that miRNAs can coordinate complex changes through simultaneous regulation of multiple targets within related biological pathways [23]. The strong regulatory connections between miR‐21 and suggested tumor suppressors PDCD4, PTEN, and RECK in our network analysis provide a mechanistic foundation for these observations, as these genes have been independently implicated in cell cycle regulation, apoptosis, and invasion suppression [24, 25, 26]. Recent advances in antisense oligonucleotide technology and miRNA sponges have made pharmacological miRNA inhibition increasingly feasible [27]. Our hypothesis‐driven network analysis, based on curated gene set of experimentally validated markers and known miR‐21 targets, consistently positions miR‐21 as the central node. This should be interpreted as descriptive network characterization rather than independent discovery. However, because miR‐21 is involved in normal physiological processes, any targeting therapeutic approach requires careful evaluation to avoid unintended effects on healthy tissues [28]. Although, our study strongly implicates miR‐21 as a key regulator, some reports have questioned its predominant role. For instance, Bilan et al. [29] observed that inhibiting miR‐21 alone had limited effects on GC cell invasion, indicating that other molecules might compensate when miR‐21 is blocked. Similarly, Chan et al. [30] reported that in certain molecular subtypes of GC, miR‐21 expression did not correlate with clinical outcomes. Several factors may explain these apparent contradictions. Studying miR‐21 alone may overlook its network‐wide functions. The molecular differences among GC subtypes may lead to context‐specific roles. In addition, our multilayer approach, which examines transcriptional, translational, and functional levels together, offers a more complete picture than studying gene expression alone. Some cancer analyses have questioned the tissue‐specific importance of the miR‐21/MMP/VEGF axis. Evidence shows varying but consistent patterns of interaction: In glioma, Gabriely et al. [31] found miR‐21 downregulates MMP inhibitors, promoting invasiveness. In cervical cancer, Yuan et al. [32] revealed a strong positive correlation between miR‐21 and VEGF expression. Hashemi M et al. [33] broadly confirms miR‐21's ability to stimulate MMP‐2 and MMP‐9 expression, enhancing tumor metastasis across multiple cancer types. Meanwhile, the specific mechanisms vary, the overall trend suggests miR‐21 consistently acts as an oncogenic regulator of the MMP/VEGF pathway.

The parallel findings from our analytical approaches strengthen the validity of our conclusions. The alignment between machine learning (identifying miR‐21 as the most important classifier), pathway analysis (showing its direct effects on tumor size), and network analysis (revealing its topological centrality) provides a strong multimethod validation of the priority of miR‐21 in this regulatory network. Furthermore, the strong correlations between MMP‐9 gene expression, protein levels, and enzymatic activity suggest the biological significance of our expression data and indicate that transcriptional regulation may play a dominant role in controlling MMP‐9 activity in GC. But our identification of MMP‐9 as a critical mediator contrasts with some studies suggesting its independent prognostic value. Although Chen et al. [34] found MMP‐9 loses significance in multivariate models, our study shows its importance lies not as an independent predictor, but as a key mediator between miR‐21 and tumor progression. These are complementary, not contradictory, insights. Study limitations include sample size requiring larger validation, cross‐sectional design limiting temporal inferences, and need for functional validation. Future studies should incorporate spatial transcriptomics and single‐cell approaches.

Limitations of this study included: our sample size (*n* = 27 patients, 54 paired samples) is modest for the complexity of our statistical models (logistic regression with six predictors, SEM). While we have employed Lasso regression, permutation testing, leave‐one‐out sensitivity analysis, and bootstrap confidence intervals to mitigate overfitting, these findings should be considered hypothesis‐generating and require validation in larger independent cohorts. As well as the functional validation (e.g., miR‐21 knockdown/overexpression, luciferase reporter assays) is required to establish causal regulatory relationships. In addition, while our random forest classifier achieved a cross‐validated AUC of 0.86 (95% CI: 0.739–0.863), the wide confidence interval reflects our modest sample size. Independent validation in a larger cohort is required before any diagnostic utility can be claimed. We have therefore refrained from suggesting clinical diagnostic applications.

In conclusion, our integrated biology systems analysis supports the view that miR‐21 may act as a key regulator. The convergent evidence from multiple analytical approaches provides a robust foundation for this model and offers novel insights into GC pathogenesis. From a biological perspective, it illustrates the power of biology systems approaches to reveal organizational principles in complex diseases. Future research should focus on validating these findings in larger cohorts, elucidating the dynamic behavior of this network during disease progression, and developing targeted interventions based on these insights. As biology systems advances, it is transforming our view of cancer from a simple list of broken molecules to a complex concept, a disrupted biological system. This shift may help to reveal more effective and personalized diagnosis and treatments for patients.

## Author Contributions

**Fatemeh Babajani:** conceptualization. **Hadi Mozafari:** conceptualization.

## Funding

The authors have nothing to report.

## Ethics Statement

The present study was approved by the Research Ethical Committee of Kermanshah University of medical sciences, Kermanshah, Iran (IR.KUMS.MED.REC.1403.021).

## Conflicts of Interest

The authors declare no conflicts of interest.

## Supporting information


**Figure S1:** Calibration plot of the random forest classifier for distinguishing gastric cancer from adjacent normal tissues. The plot shows the agreement between predicted probabilities and observed outcomes across the 54 samples (27 tumor, 27 matched adjacent normal). The diagonal dashed line represents perfect calibration (predicted probability = observed proportion). The solid blue line shows the calibrated performance of the model, with the shaded area representing the 95% confidence interval. Points represent binned observations with their corresponding 95% confidence intervals. Good calibration is indicated by the proximity of the calibration curve to the diagonal line, demonstrating that the model's predicted probabilities are well‐calibrated and reliable for discriminating gastric cancer samples from normal tissues.

**Figure S2:** Permutation test for random forest classifier. Histogram of out‐of‐bag accuracies from 500 models trained on permuted cancer status labels (light blue). The red dashed line indicates the observed accuracy of the true model (85.2%). None of the permuted models achieved accuracy equal to or greater than the observed model (*p* < 0.001).

**Table S1:** KEGG pathway enrichment analysis of the targeted gene set. The analysis was performed on a curated gene list comprising experimentally validated markers (miR‐21, MMP‐2, MMP‐9, VEGFA) along with experimentally confirmed targets of miR‐21 (PDCD4, PTEN, RECK, TIMP3, and SPRY2) retrieved from miRTarBase (version 9.0). Enrichment analysis was conducted using the clusterProfiler package in R with hypergeometric test and Benjamini‐Hochberg false discovery rate (FDR) correction. Pathways with FDR < 0.05 were considered statistically significant, except for VEGF signaling pathway (FDR = 0.085) which is shown as nominally significant. The table displays pathway names, KEGG identifiers, gene ratios (proportion of input genes mapping to each pathway), raw *p* values, FDR‐adjusted *p* values, and the specific genes involved in each pathway. The MicroRNAs in cancer pathway (hsa05206) showed the strongest enrichment (FDR = 2.09 × 10^−7^), followed by Bladder cancer (FDR = 0.00149) and Proteoglycans in cancer (FDR = 0.00866). These results contextualize our experimentally validated markers within established cancer‐related signaling networks.

**Data Set: S1.** KEGG pathway enrichment analysis.

## Data Availability

The data that support the findings of this study are available from the corresponding author upon reasonable request.
