# Supplementary figures and images for: Integrated Network Analysis Suggests an miR‐21/MMP/VEGF‐Associated Regulatory Axis in Gastric Cancer
Source: Cancer Rep (Hoboken). 2026 Jul 31;9(8):e70634. doi: 10.1002/cnr2.70634 (PMC13428076; doi:10.1002/cnr2.70634)

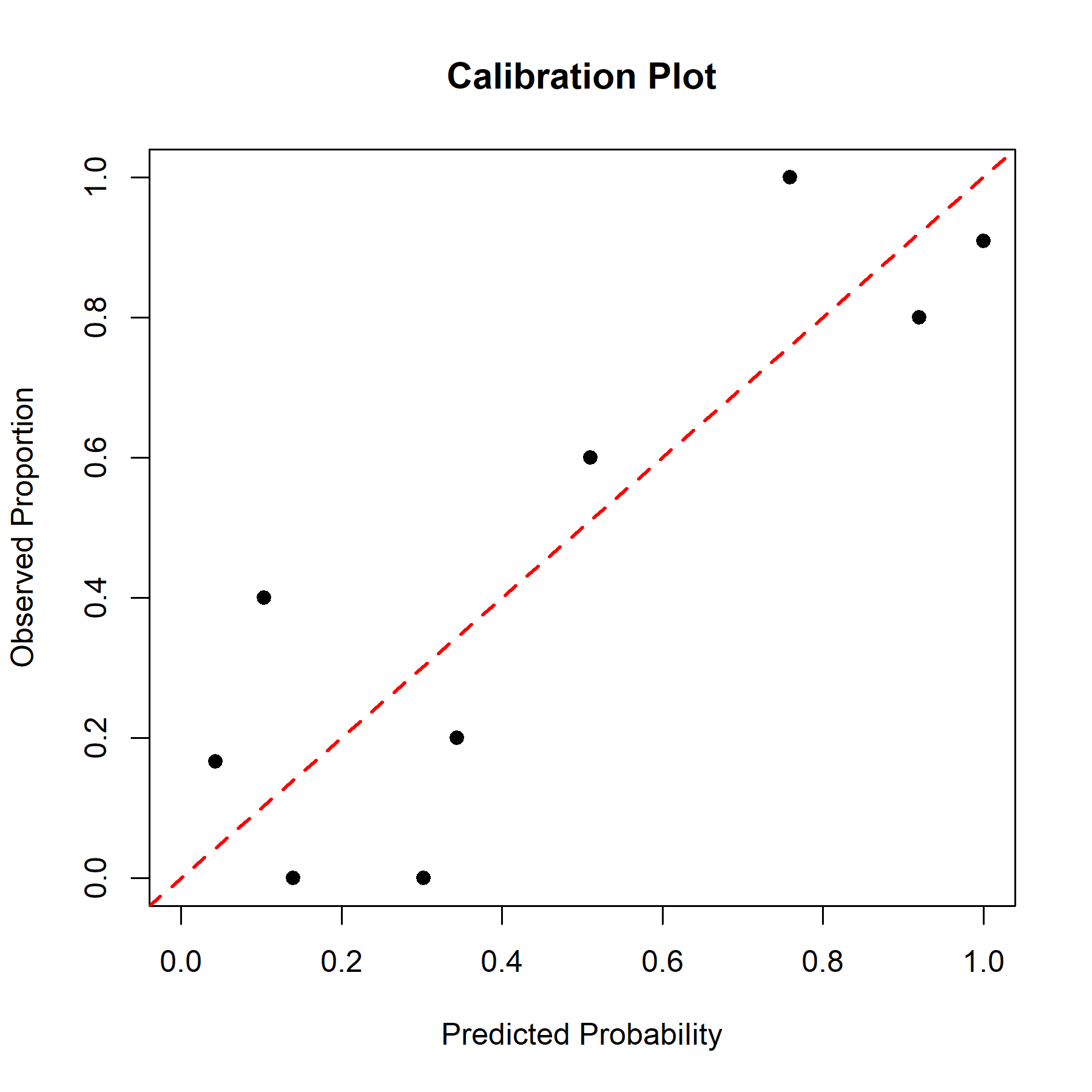

Supplement: Supplementary file 1 — Figure S1: Calibration plot of the random forest classifier for distinguishing gastric cancer from adjacent normal tissues. The plot shows the agreement between predicted probabilities and observed outcomes across the 54 samples (27 tumor, 27 matched adjacent normal). The diagonal dashed line represents perfect calibration (predicted probability = observed proportion). The solid blue line shows the calibrated performance of the model, with the shaded area representing the 95% confidence interval. Points represent binned observations with their corresponding 95% confidence intervals. Good calibration is indicated by the proximity of the calibration curve to the diagonal line, demonstrating that the model's predicted probabilities are well‐calibrated and reliable for discriminating gastric cancer samples from normal tissues. [file CNR2-9-e70634-s002.docx]
