# Supplementary material for: Integrated Network Analysis Suggests an miR‐21/MMP/VEGF‐Associated Regulatory Axis in Gastric Cancer
Source: Cancer Rep (Hoboken). 2026 Jul 31;9(8):e70634. doi: 10.1002/cnr2.70634 (PMC13428076; doi:10.1002/cnr2.70634)
Supplement: Supplementary file 2 — Figure S2: Permutation test for random forest classifier. Histogram of out‐of‐bag accuracies from 500 models trained on permuted cancer status labels (light blue). The red dashed line indicates the observed accuracy of the true model (85.2%). None of the permuted models achieved accuracy equal to or greater than the observed model (p < 0.001). [file CNR2-9-e70634-s004.docx]

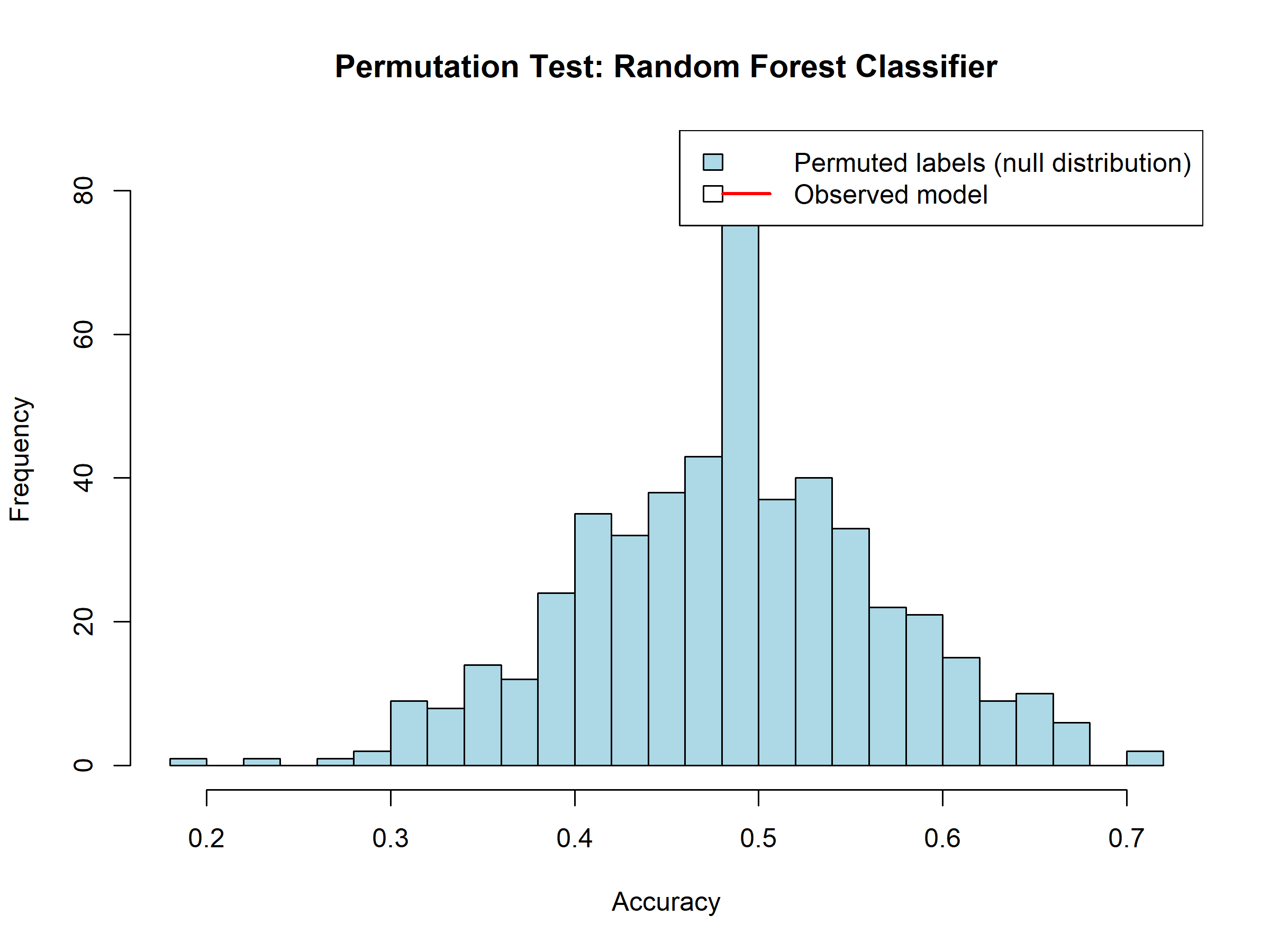


**Supplementary Figure S2. Permutation test for random forest classifier.** Histogram of out-of-bag accuracies from 500 models trained on permuted cancer status labels (light blue). The red dashed line indicates the observed accuracy of the true model (85.2%). None of the permuted models achieved accuracy equal to or greater than the observed model (p < 0.001).
