# Supplementary material for: Integrated Network Analysis Suggests an miR‐21/MMP/VEGF‐Associated Regulatory Axis in Gastric Cancer
Source: Cancer Rep (Hoboken). 2026 Jul 31;9(8):e70634. doi: 10.1002/cnr2.70634 (PMC13428076; doi:10.1002/cnr2.70634)
Supplement: Supplementary file 4 — Data Set: S1. KEGG pathway enrichment analysis. [file CNR2-9-e70634-s001.pdf]

==

KEGG Pathway Enrichment Analysis

==

Supplementary File S1

library(clusterProfiler)

Entrez ID

MIR21: 406991

MMP2: 4313

MMP9: 4318

VEGFA: 7422

PDCD4: 27250

PTEN: 5728

RECK: 8434

TIMP3: 7078

SPRY2: 10253

gene_list <- c(“406991”, “4313”, “4318”, “7422”, “27250”, “5728”, “8434”, “7078”, “10253”)

KEGG enrichment

kegg_result <- enrichKEGG(gene = gene_list, organism = “hsa”, pvalueCutoff = 0.05, pAdjustMethod = “BH”)

print(kegg_result)

write.csv(kegg_result, “kegg_enrichment_results.csv”)
